# Non-Destructive Testing of a Sport Tribune under Synchronized Crowd-Induced Excitation Using Vibration Analysis

**DOI:** 10.3390/ma12132148

**Published:** 2019-07-04

**Authors:** Karol Grębowski, Magdalena Rucka, Krzysztof Wilde

**Affiliations:** 1 Department of Technical Fundamentals of Architectural Design, Faculty of Architecture, Gdansk University of Technology, Narutowicza 11/12, 80-233 Gdansk, Poland; 2 Department of Mechanics of Materials and Structures, Faculty of Civil and Environmental Engineering, Gdansk University of Technology, Narutowicza 11/12, 80-233 Gdansk, Poland

**Keywords:** non-destructive testing, reinforced concrete grandstand stadium, vibration analysis, crowd-induced excitation, structural tuning

## Abstract

This paper presents the concept of repairing the stand of a motorbike speedway stadium. The synchronized dancing of fans cheering during a meeting brought the stand into excessive resonance. The main goal of this research was to propose a method for the structural tuning of stadium stands. Non-destructive testing by vibration methods was conducted on a selected stand segment, the structure of which recurred on the remaining stadium segments. Through experiments, we determined the vibration forms throughout the stand, taking into account the dynamic impact of fans. Numerical analyses were performed on the 3-D finite element method (FEM) stadium model to identify the dynamic jump load function. The results obtained on the basis of sensitivity tests using the finite element method allowed the tuning of the stadium structure to successfully meet the requirements of the serviceability limit state.

## 1. Introduction

Stadiums are sport objects that require a particular level of attention to ensure human security. During the last century, many tragedies have been caused mainly by negligence at the stage of designing the structure. Nowadays, many structural projects are sought to be optimized. The results are slender, more effective and economical objects; however, they are often sensitive to dynamic impacts.

Since 1902, more than 20 construction disasters of stadium stands have taken place around the world [1,2]. These range from the stand collapse at Ibrox Park stadium in Glasgow (Scotland) on April 5, 1902 during the Scotland vs. England match, which resulted in 26 dead and 550 injured supporters, to the tragedy that took place on November 26, 2007 at the Salvador stadium (Brazil) during the Bahia vs. Vila Nova match, which resulted in eight deaths and 150 injured due to the collapse of the stand.

Strong vibrations caused by moving people mainly occur in structures with low rigidity and mass. Stadiums are an example of this type of structure. High dynamic loads occur, caused by jumping fans cheering their teams. This type of load is often omitted during the structure design, because procedures in current standards or guidelines regarding the jump load are limited.

In recent years, many studies have been devoted to the dynamic impact of stadium structures. Development in sensor technology, through the supply of adapted vibration transducers (low-level vibration, high-level/shock vibration, near-static sensitivity, etc.) in conjunction with the dissemination of digital processing algorithms, has led to a notable increase in the study of vibrations caused by crowd-induced excitation in structures [3]. Modal identification results can be used as part of the vibration reduction methodologies [4]. In [5], it was found that crowd occupation can significantly alter the modal properties of a stadium, and that the changes vary according to crowd configuration. A large number of publications on structural response and stadium vibration resistance due to crowd-induced loads have shown that the dynamic response of stadiums depends not only on the basic mechanical characteristics, i.e., mass, stiffness and damping, but also on the nature of the load, which can become complex in the case of jumping supporters (e.g., [6]). In order to limit the vibrations of the structure, one of the countermeasures is frequency tuning a structure [7,8]. A comprehensive analysis of the literature on dynamic performance tests of existing stadium structures was conducted in [9]. It was concluded that the available knowledge on this subject is not yet sufficiently advanced and thus jump load is not currently included in most design standards. Therefore, the dynamic identification based on non-destructive testing methods is increasingly popular and effective in civil engineering research. Several literature studies have shown how non-destructive testing and structural health monitoring can be efficiently used to assess the durability of reinforced concrete structures [8,9,10,11,12,13,14,15].

An example of stand failure due to the dynamic impact of fans is the case of the Swiss Krono Arena motorbike speedway stadium in Zielona Góra (Poland). The new stand was built in 2009–2010. In July 2010, it was put into service. The stand is a reinforced concrete structure that is used in a very specific way. Speedway meetings are a sport discipline that arouse great emotions and interest among supporters. One of the basic forms of team cheering at the Swiss Krono Arena is the so-called “Labado dance”. The dance is considered as almost a hymn among speedway fans. In this dance, fans put their hands on their neighbor’s shoulders and in the rhythm of the animator’s drumming, they jump simultaneously to the song “(...) we dance labado, labado, (...)”. In 2010 it was noticed that the fans’ dance causes an increase in the vibrations of the structure, which led to a discussion on the safety of using the new stand. Research on the assessment of the harmfulness of vibrations was carried out in 2010–2011 by a team from University of Zielona Góra, and in the years 2011–2012 by a team from Gdańsk University of Technology. The Labado dance appeared to be dangerous for the stand’s structure, because by jumping fans caused a vertical periodic force, which when synchronized movements of a large number of people could lead to resonant vibrations, possibly ending with the collapse of the structure. A large number of people generating regular dynamic excitations could be able to destroy any bridge or reinforced concrete stadium stand, which is why this phenomenon is considered vandalism.

This paper presents non-destructive testing of a sport facility subjected to dynamic interactions in the form of jumping fans. Experimental tests were conducted in the field on the Swiss Krono Arena speedway stadium in Zielona Góra. Later on, numerical simulations were performed to determine the dynamic characteristics of the structure. Validation was carried out by comparing the numerical model with the results obtained in the field. After the validation of the numerical model, the identification of the jump load dynamic function was determined based on laboratory tests. Lastly, the stadium stand tuning model was re-tested in situ after structure strengthening.

## 2. Theoretical Background of the Experimental Modal Analysis

**Figure 1:**
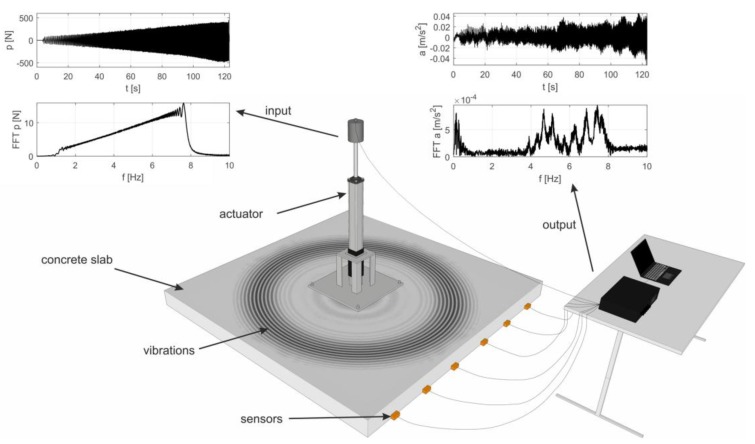
Scheme of the experimental modal analysis technique.

Modal analysis is a method of determining dynamic properties of structures (i.e., natural frequencies, modal shapes and damping coefficients) under vibrational excitation. The analysis involves the registration of vibrational signals and the application of different signal processing techniques. In general, two types of modal analysis can be distinguished: operational modal analysis (OMA) [16,17,18,19] and experimental modal analysis (EMA) [20,21,22,23,24]. Environmental excitations (e.g., wind, sea waves, micro-seismic vibrations, traffic loads, etc.) are used in the OMA technique, and only the structure’s response is recorded. In the case of EMA, both excitation and response are measured. The most common approach in EMA is the impulse test, which is based on the excitation of the structure using a modal hammer and the harmonic test, in which electromechanical or piezoelectric actuators are usually applied (Figure 1).

First, the input force *p*(*t*) and output displacement *u*(*t*) time signals are measured in EMA, and then they are transformed into the frequency domain by Fourier transform, resulting in *P*(*ω*) and *U*(*ω*) signals (Figure 1). The next step is the calculation of the frequency response function (FRF) in all measured points, which is a ratio of the *j*-th output and the *k*-th input:

(1) $$H_{jk}(\omega )=\frac{U_{j}(\omega )}{P_{j}(\omega )}.$$

In the practical application of EMA, acceleration signals are often measured instead of displacement signals, as they are more convenient in analytical works. Then, the accelerance function can be defined as:

(2) $$A_{jk}(\omega )=\frac{{\ddot{U}}_{j}(\omega )}{P_{j}(\omega )}.$$

Both the receptance and accelerance functions describe the same dynamic properties of the system and the relationship between them is given by:

(3) $$A_{jk}(\omega )=-\omega^{2}H_{jk}(\omega ).$$

Based on the frequency response functions (FRFs), resonance frequencies, mode shapes and damping coefficients can be identified (e.g., [25,26]).

## 3. Experimental Dynamic Identification of the Grandstand

### 3.1. Description of the Structure

**Figure 2:**
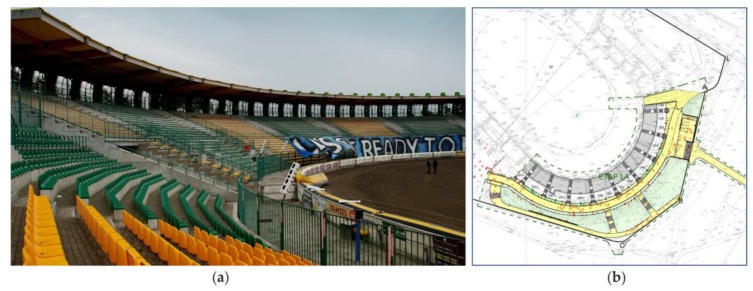
Swiss Krono Arena: (**a**) the view on the grandstand, (**b**) the location of the grandstand on the land development plan.

The Swiss Krono Arena motorbike speedway stadium is located in Zielona Góra (Poland). The grandstand was erected in 2009–2010 and its geometry is a circular sector with a center angle of 150° (Figure 2).

The stand consists of a reinforced concrete structure in which prefabricated under-seat beams rest on the main beams of reinforced concrete girders. The main girder is composed of two parts and it is supported on three reinforced concrete columns. The first part of the main girder (BR1) is an inclined simply supported beam, located in the bottom part of the stand. The second part of the girder (BR2), with a variable cross-sectional height, also works as a simply supported beam, and it is finished with a cantilever beam located in the upper part of the stand.

**Figure 3:**
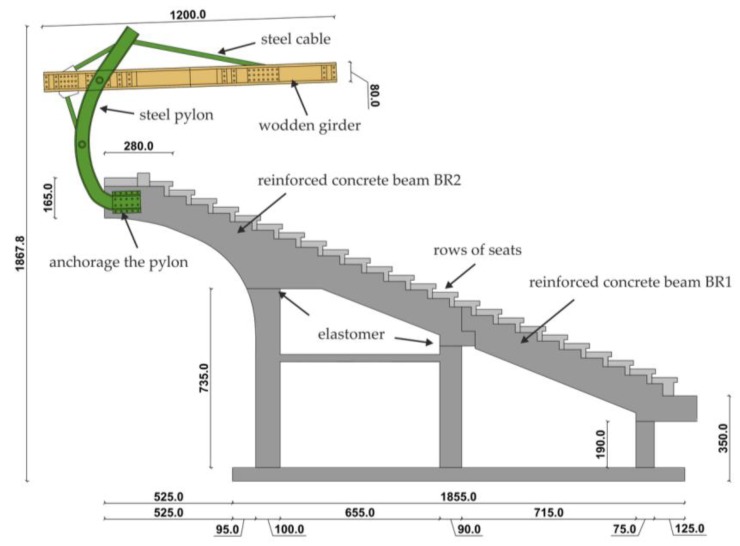
Cross-section of the stand (dimensions in cm).

Both girders are connected to each other by an articulated joint. The main beams are based on columns via elastomeric pads. Steel anchors prevent horizontal displacement of the main beams against columns. The columns and the monolithic wall supporting the main girder are located on reinforced concrete strip foundations. The walls of additional objects are made of silicate brick, while the stairs structure is made as a prefabricated reinforced concrete element (Figure 3). 

The roof structure over the stand consists of three elements: steel columns, which are attached to the cantilever ends of the main load-bearing girder; the roof, which consists of wooden girders; and purlins, to which the covering trapezoidal sheeting and cables are attached. The cables connect the wooden roof structure with steel columns. The steel supporting columns of the roof structure are braced with steel braces. Roof bracings are also used in the wooden roof structure.

### 3.2. Test Procedure and Vibration Measurement

**Figure 4:**
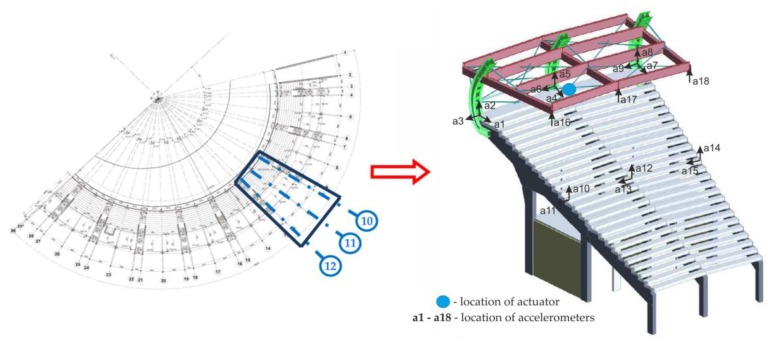
The stand section selected for field tests.

The measurements were conducted on a selected section of the stand, as shown in Figure 4. The experimental program included a sweep sine test and two types of people-induced vibrations, namely single jumps and synchronous Labado dancing.

Triaxial piezoelectric accelerometers 356B18 (PCB Piezotronics, Inc., Depew, NY, USA) were used for the measurement of vibrations. They were attached to both the concrete part of the stand as well as to the roof structure. Vibrations signals were registered at nine points (Figure 4), in one, two and three directions. In total, 18 acceleration signals were acquired (a1 to a18). Data acquisition and signal conditioning were performed by the LMS SCADAS portable system (Siemens, Leuven, Belgium). The sampling frequency was set as 256 Hz.

**Figure 5:**
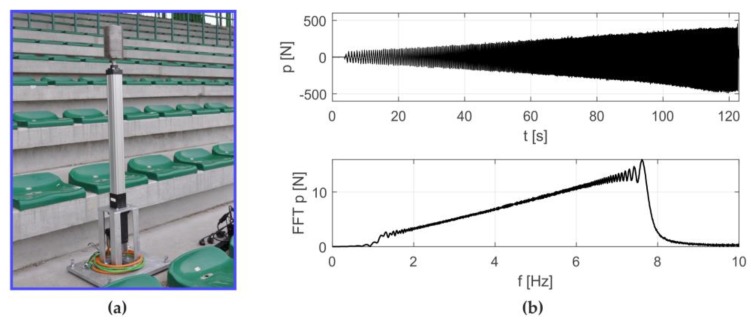
Experimental setup: (**a**) electromechanical actuator, (**b**) excitation signal in the time and frequency domains.

In the first stage, the experimental modal analysis was conducted. The harmonic load was excited by means of the electromechanical actuator shown in Figure 5a. The excitation signal, created by an arbitrary signal generator, was a sweep sine of a smoothly adjustable frequency from 1 Hz to 8 Hz (Figure 5b).

**Figure 6:**
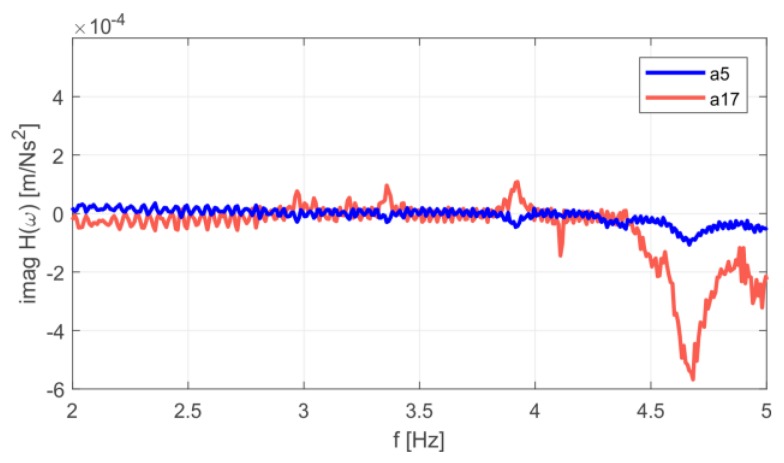
Imaginary parts of the frequency response function (FRF) for acceleration signals a5 (girder) and a17 (roof).

**Figure 7:**
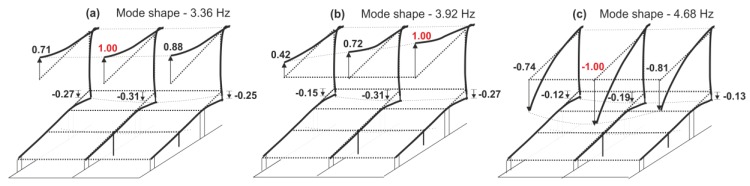
Identified mode shapes and natural vibrations: (**a**) 3.36 Hz, (**b**) 3.92 Hz, (**c**) 4.68 Hz.

Dynamic parameters were determined based on the frequency response functions, according to procedure described in Section 2. The imaginary parts of the FRFs for signals registered on the girder and on the roof are given in Figure 6. Several peaks can be distinguished. The experiment allowed the identification of three natural frequencies in the range from 2 to 5 Hz (Figure 6). Their values were: 3.36 Hz, 3.92 Hz and 4.68 Hz. Experimentally determined mode shapes are presented in Figure 7. The directions and values of displacements for particular degrees of freedom are shown by means of vectors and numerical values. The shapes of modes of the girder and roof are similar, wherein the amplitude for the roof is much larger. The dominant vibration types for each identified form are vertical mode shapes. The main displacements of the girder and roof occurred as a result of vertical motion.

The next step included the non-destructive evaluation of the stand throughout the analysis of the forced vibrations. The impact of the real dynamic forces was implemented thanks to the fans who, at the request of the club authorities, came to participate in the measurements. About 400–450 people participated in the research. For the purpose of the research, the fans presented two forms of active support: the so-called Scotland-type jumps and the Labado dance. By making the Scotland-type jumps, the fans performed single jumps to a drum beat. They started very slowly and finished with asynchronous jumps. In the Labado dance, almost all fans performed synchronous jumps with a constant frequency.

**Figure 8:**
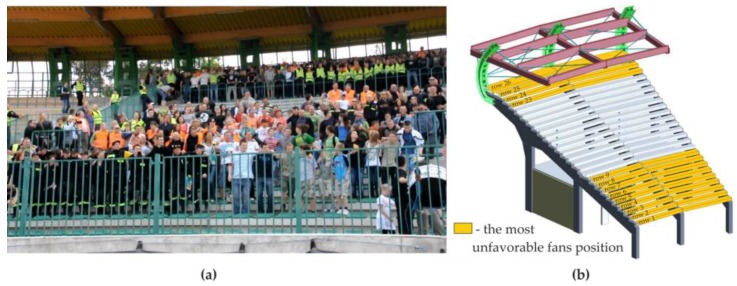
Dynamic tests with fans: (**a**) experimental setup, (**b**) scheme showing the arrangement of people during tests (orange color—rows indicating the most unfavorable fan positions).

During the research, one of the most important tasks was to find the most unfavorable load combination. Different combinations of fans' arrangement on stadium stand rows were tested. One of the most unfavorable combinations, resulting in the greatest vibrations of the cantilever part of the stand, was selected for the purpose of the tests. Figure 8 presents the most unfavorable setting in which the dancing fans are located at the bottom of the stand (beam BR1) and on the upper part of the stand (four rows at the cantilever part of beam BR2).

**Figure 9:**
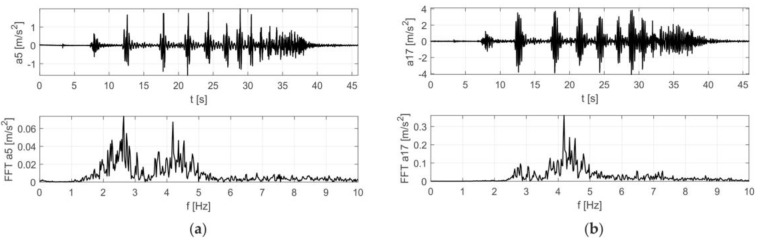
Accelerations registered during dynamic tests with fans (Scotland-type jumps) at: (**a**) girder, (**b**) roof.

**Figure 10:**
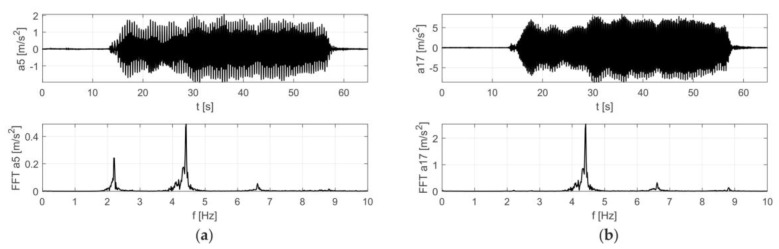
Accelerations registered during dynamic tests with fans (Labado dance) at: (**a**) girder, (**b**) roof.

The results of the in situ tests are shown in Figure 9 and Figure 10 for the Scotland-type jumps and the Labado dance, respectively. In the case of the Scotland-type jumps, many jump events are visible, with decreasing time between individual jumps. In the Fourier transformation results, two wide peaks are visible, the first around 2.5 Hz and the second around 4.5 Hz. The Labado dance, in which jumps were more regular, resulted in narrower peaks concentrated at 2.2 and 4.4 Hz.

Based on the obtained results, it was found that the frequency of vibrations of the end of the cantilever girder was equal to 2.2 Hz, coinciding with the frequency of the exciting force, which was caused by jumping supporters. The end of the roof structure vibrated at 4.4 Hz, which means that the roof vibrated twice as fast as the end of the girder.

## 4. Finite Element Method Modeling

### 4.1. Identification of the Jump Load Function

**Figure 11:**
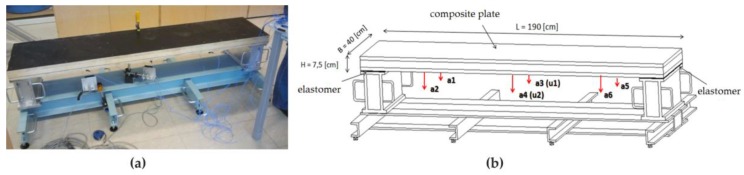
Experimental setup: (**a**) general view, (**b**) geometry of the tested plate and the location of points for measurement of accelerations (a1 to a5) and displacements (u1, u2).

Experimental tests were conducted in the laboratory of the Gdańsk University of Technology in order to identify the jump load function. The test object was a composite plate with dimensions of 190 cm × 7.5 cm × 40 cm (Figure 11a). The plate was made of two sheets of poplar plywood of a thickness of 2 cm. Between them, C 20 class wood with a 3.5 cm thickness was inserted. Material parameters adopted for testing were: bending strength 20 MPa, stretching along fibers 12 MPa, compression along fibers 19 MPa, modulus of elasticity along fibers 10 GPa, average density 330 kg/m^3^. The plate was placed on supports using elastomer pads with dimensions of 7 cm × 40 cm × 0.5 cm.

**Figure 12:**
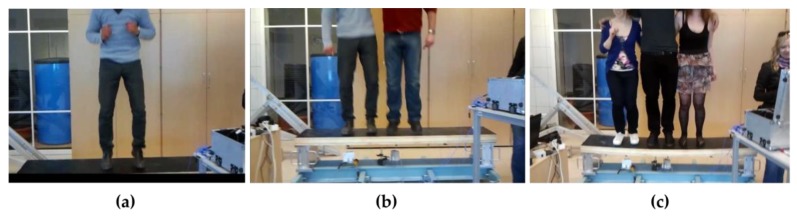
Implementation of excited vibrations in the form of rhythmical jumps of (**a**) one person, (**b**) two people, (**c**) three people.

The plate was subjected to dynamic tests. The acceleration measurements (a1 to a6) were performed at six points by means of the PCB accelerometers model 356A16, while displacement measurements were taken at two points (u1 to u2) by means of optoNCDT 1302 laser sensors, as shown in Figure 11b. The LMS SCADAS vibration measurement system was used to record the time histories. The research agenda included measurements of free vibrations as well as measurements of forced vibrations in the form of synchronized jumps performed by one, two and three people (Figure 12). 

**Figure 13:**
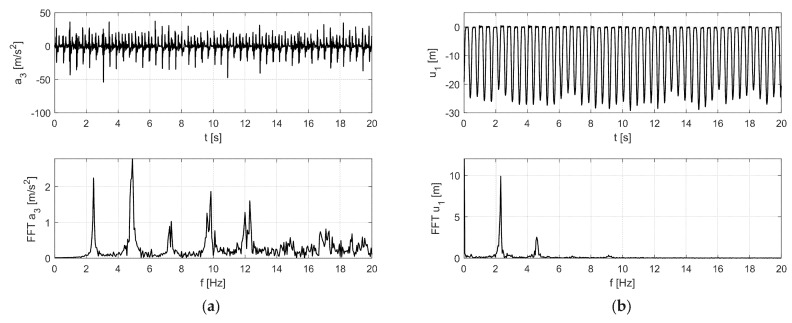
Results of vibrations in the form of (**a**) accelerations and (**b**) displacements recorded during the jumps of three people.

The first natural frequency of the plate was identified using a typical impact test. Its value was 26 Hz. Figure 13 present the results of vibrations in the form of accelerations and displacements recorded during the jumps of three people. Apart from the jump frequency, further components of harmonically excited vibrations are visible in the Fourier transform diagrams. Eight harmonic components are visible on the Fourier transforms diagrams on acceleration, while the Fourier transforms of displacement signals show four harmonic components.

**Figure 14:**
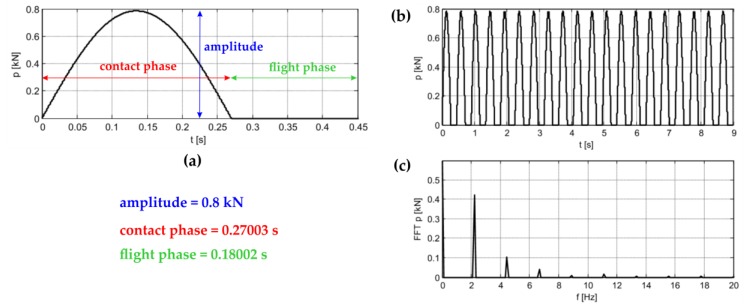
Labado function: (**a**) one jump, (**b**) 20 jumps, (**c**) Fourier transformation of the Labado function.

The dynamic impact function during the jumping was described by means of an impulse shaped in half of the sinus function period form. The loading cycle for the Labado dance includes the flight phase and the contact phase. On the basis of the measurement results, the duration of the periodic force full period was identified as 0.45005 s, which is composed of: the phase of load contact with the plate equal to 0.27003 s and the flight phase equal to 0.18002 s. The shape of a single dance cycle is shown in Figure 14a. The in situ tests indicated the jump frequency to be 2.2 Hz. The amplitude of the load function was determined as the average value of the weight of fans participating in the experiment, and was equal to approximately 0.78 kN.

Figure 14b,c shows excitation over time during 20 rhythmic jumps and the corresponding Fourier transform. The Fourier transform graph proves that, in addition to the 2.2 Hz jump frequency, further harmonics are present in the spectrum. The occurrence of higher harmonics is characteristic of this periodic signal. Higher harmonic components are also present on transforms from the experimental data.

**Figure 15:**
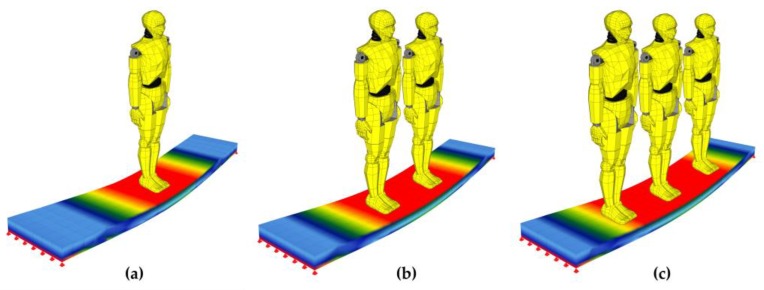
Numerical simulations of the implementation of excited vibrations in the form of rhythmic jumps performed by: (**a**) one person, (**b**) two people, (**c**) three people.

In order to validate the laboratory tests results, numerical simulations for the composite plate were performed. The calculated plate length was set as 183 cm. It was assumed that the plate works in a simply supported scheme. In the discretization process, the slab was divided into 504 solid elements in accordance with the results obtained during the convergence division analysis. Numerical calculations were performed for excitation caused by one, two and three people (Figure 15).

**Figure 16:**
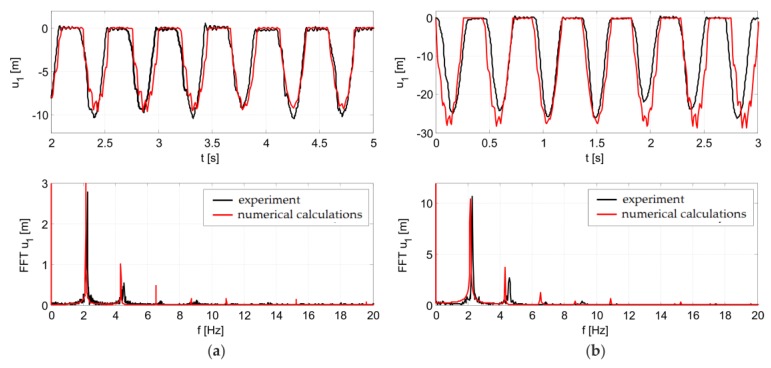
Numerical model validation results for jumps performed by: (**a**) one person, (**b**) three people.

Figure 16 presents a comparison of the numerical and experimental results in the form of displacement in time, registered at the middle of the span, and their Fourier transforms for the cases of one and three people jumping. In the flight phase, plate vibrations occurred at a frequency of 26 Hz for both the experimental and numerical results. Slight phase shifts between the experimental and numerical graphs can be observed. The reason for the shifts lies in the fact that the real jumps were not perfectly repetitive. A very good experiment consistency of the numerical simulations was obtained, which proves the correctness of the assumed load model for the Labado dance. The excitation frequency was approximately 2.2 Hz during both the experimental and numerical tests. Later in the article, the obtained Labado dance load model is used to simulate vibrations and structure tuning of the stand at the Swiss Krono Arena speedway stadium.

### 4.2. Numerical Analysis

**Figure 17:**
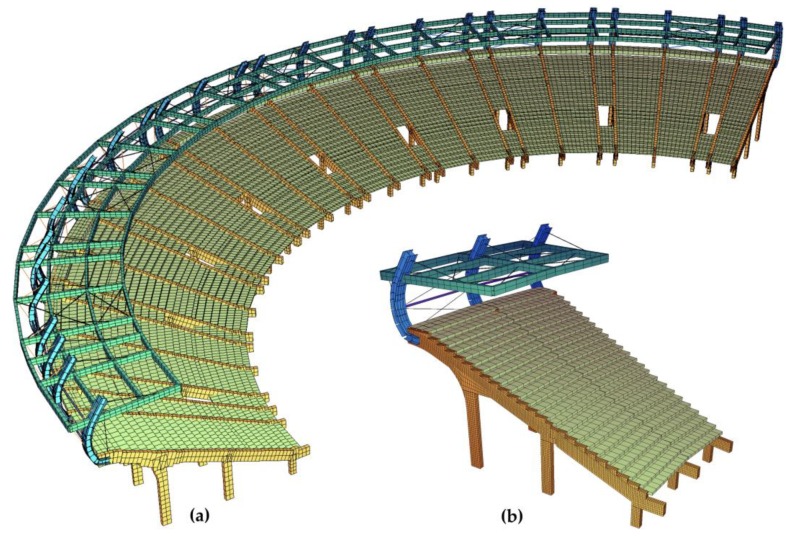
(**a**) Numerical model of the stadium, (**b**) numerical model of a single section of the stand.

The creation of the 3-D FEM model of the stand at the Swiss Krono Arena stadium was implemented in two stages with the use of the commercial FEM software SOFiSTiK (SOFiSTiK AG, Oberschleißheim, Germany). In the first stage, models of the main structural parts of the stand were performed using solid elements (Figure 17b), while in the second stage they were performed with the use of beam elements (Figure 17a). The construction of beam models was verified based on the results of numerical analyses carried out on solid models, due to which a very high consistency was obtained between the results of the static and dynamic tests. The FEM calculation model of the entire prefabricated 3-D stadium contains approximately 3008 beam elements and 2001 nodes, in accordance with the results collected during the convergence analysis. Substitute cross-sections were applied in the beam model, in which the cross-section of concrete and steel was replaced with a representative cross-section of a homogeneous material. The under-seat beams of the stand were modeled as beam elements. The columns were articulately connected with the girders, and an articulated joint can also be found at the connection of the two main girders. Glued laminated timber girders were modeled as beam elements, and steel bracings and bar elements model the truss elements. In models that consider various stand strengthening methods, the tensile elements are modeled using tensile elements (cable) taking into account the prestressing force. Non-structural elements of significant weight (e.g., non-structural steel columns) were modeled as concentrated masses applied at the place of mounting. The material parameters taken for the purpose of the numerical analysis were adopted in accordance with the construction and structural design.

**Figure 18:**
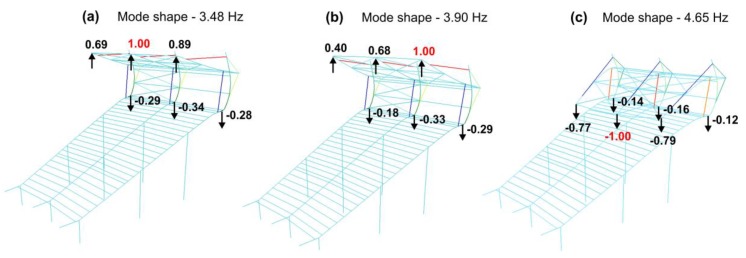
Mode shapes and frequencies of natural vibrations of a single stand section obtained from numerical simulations: (**a**) 3.48 Hz, (**b**) 3.90 Hz, (**c**) 4.65 Hz.

The eigenvalues were calculated at the beginning. Thirty eigenvalues of the system were calculated. The total mass contributions in the dynamic response were more than 90% in directions: X (longitudinal), Y (transverse) and Z (vertical), which complied with the standard recommendations. The first three eigenvalues and their corresponding natural vibration frequencies determined on the basis of modal analysis are presented below (Figure 18).

The determined basic natural vibration frequency of the stand construction with a roof and without strengthening was used to simulate forced oscillations and to determine the degree of stiffness reduction of the concrete cross-section due to scratches. For the purpose of the analysis, the structure was loaded with its own weight and an evenly distributed load on the stands of 8.0 kN/m^2^, in accordance with PN-EN-1991-1-1 [27]. It was assumed that this load changes harmonically according the to the relation p(t)=psin(ωt), where the amplitude *p* = 8.0 kN/m^2^ and the circular frequency ω = 15.71 rad/s (which corresponds to the frequency *f* = 2.3 Hz). It has been assumed that the load of such nature lasts *t* = 60 s, the same as in the experiment.

**Figure 19:**
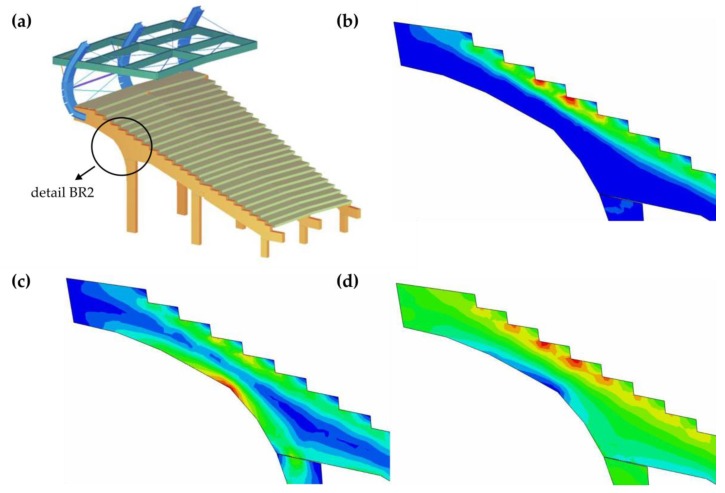
(**a**) Placement of the highest stress concentrations in the main girder of the stadium stand. (**b**) von Mises map of stresses in the BR2-reinforced concrete girder area (red color—maximum stress equal to 10 MPa). (**c**) Map of the main tensile stresses within the BR2-reinforced concrete girder (red color—maximum tensile stress equal to 8 MPa). (**d**) Map of the horizontal normal stress s11 within the BR2-reinforced concrete girder (red color—maximum tensile stress equal to 8 MPa, blue color—maximum compressive stress equal to 8 MPa).

Figure 19 shows the stresses in the BR2 girder. Figure 19b shows equivalent von Mises stresses, Figure 19c presents the main tensile stresses and Figure 19d demonstrates the normal horizontal stresses in the girder plane (in the global coordinate system). The main tensile stress in the upper part of the BR2 beam was greater than the concrete tensile strength, i.e., $\sigma_{1}=8\;\mathrm{MPa}>\sigma_{cr}=f_{ctm}=3.2\;\mathrm{MPa}$, which means that the reinforced concrete section becomes scratched.

The decrease in the stiffness of the scratched girder was equal to about 50%. The decrease of the stiffness of the reinforced concrete section after scratching was determined on the basis of the obtained stress distribution in the prefabricated beam supporting the stands. During the numerical simulation, the basic natural vibration frequencies of the structure were determined, taking into account the stiffness reduction of the reinforced concrete cross-section due to scratching. 

**Figure 20:**
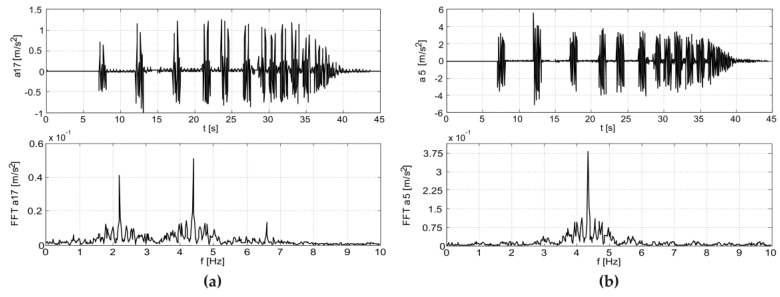
Results of numerical simulations from the excitation of vibrations by jumping fans (Scotland-type jumps): (**a**) girder, (**b**) roof.

**Figure 21:**
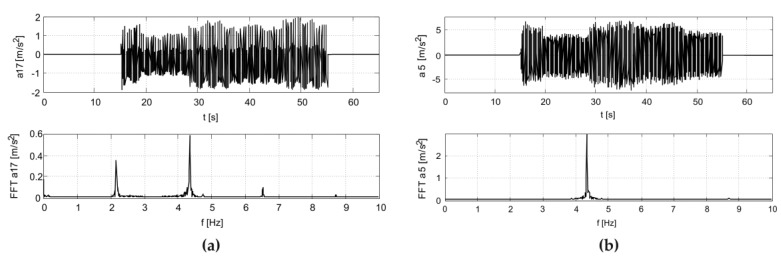
Results of numerical simulations from the excited vibrations caused by jumping fans (Labado dance): (**a**) girder, (**b**) roof.

In the further stage of the numerical analysis, the stadium structure was loaded with jumping fans. Each of the fans was replaced by a single concentrated force with a load value of 0.8 kN, as this load corresponded to the average human weight. A jump function determined during experimental research was assigned to every concentrated force. The duration time was equal to 40 s. The forces were set in the most unfavorable load combination, i.e., at the bottom of the stand (chairs on the whole BR1 beam width) and on the upper part of the stand (four rows above the cantilever section). Simulations of stand vibrations for the excitation form of non-synchronized Scotland-type jumps were carried out at the beginning (Figure 20). Afterwards, an analysis for the form of the synchronized Labado dance was performed (Figure 21). 

On the basis of the results obtained during non-destructive tests and numerical simulations, it was concluded that as a result of dynamic interactions, the phenomenon of higher harmonic resonance occurred on the stand and the roof structure. The vibrations frequency at the end of the roof was equal to approximately 4.7 Hz, which coincides with the second harmonic of the excitation force, i.e., 4.4 Hz, caused by jumping fans. The form and dynamic characteristics of the Labado dance can be considered as vandalism. A large number of jumping fans with their synchronized movements drives the tribune structure’s vibrations, and their regular dance could cause the collapse of the entire object. The results of non-destructive tests and numerical simulations showed a significant excess of the serviceability limit state.

## 5. Structural Tuning

**Figure 22:**
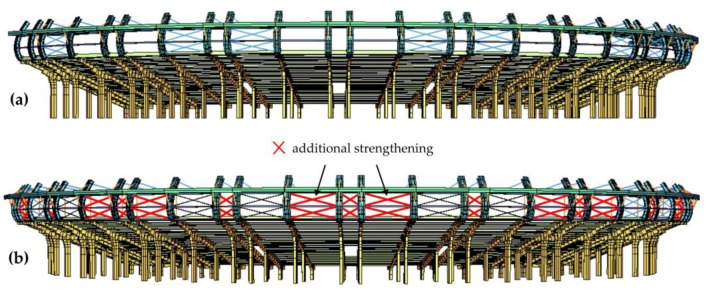
Additional wall bracings (**a**) before structural tuning, (**b**) after structural tuning.

**Figure 23:**
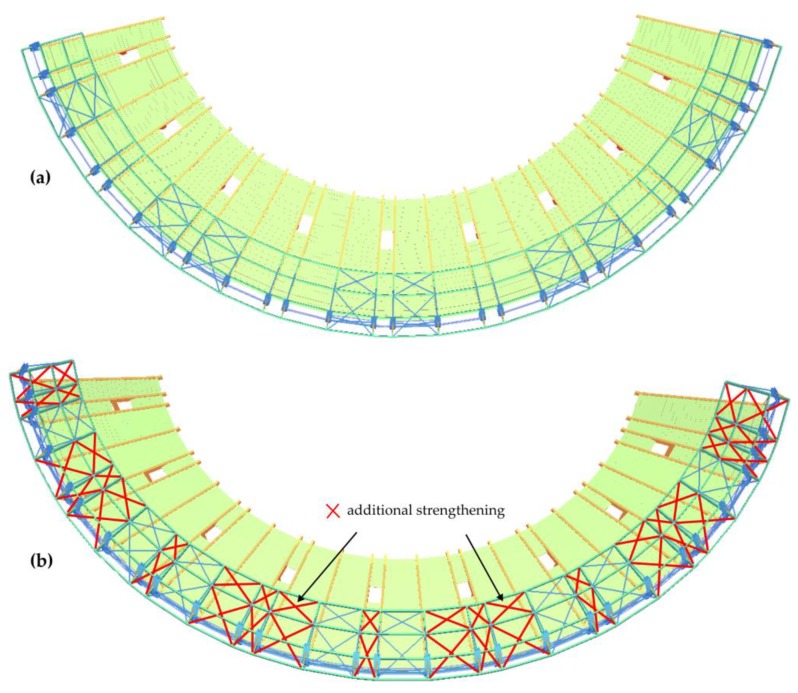
Additional roof bracings (**a**) before structural tuning, (**b**) after structural tuning.

An original method of stand structural tuning was proposed in response to the excess of the serviceability limit state. This method assumed the implementation of wall bracings stiffening the steel roofing columns, as well as roof bracings on the wooden roof structure (Figure 22 and Figure 23). The main goal of the concept of bracing of all columns and roof girders was to significantly limit the free end of the wooden roof vibration amplitudes, and hence reduce the concrete cantilever displacements. The bracing cross-sections were the same as those of the bracings already installed in the stand structure. The bracings were made from Macalloy bar rods with a yield point of 520 MPa and tensile strength of 690 MPa.

**Figure 24:**
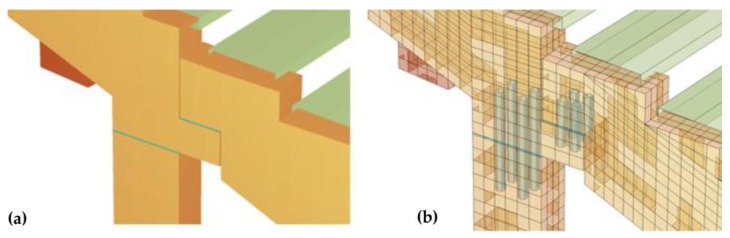
Detailed view of the stand's load-bearing beams: (**a**) support, (**b**) connection.

The stand FEM model with additional roof bracings and vertical bracings was implemented in SOFiSTiK software. The beam stand model was made on the basis of the object design documentation (Figure 24). The basic model parameters were determined on the basis of detailed models with the use of solid elements, in which the location of reinforcement bars and details of beams support, such as elastomer pads and anchors, were taken into account. The models include the scratching of concrete elements by reducing the value of the concrete modulus of elasticity. The FEM model parameters were updated on the basis of the obtained measurement data.

**Figure 25:**
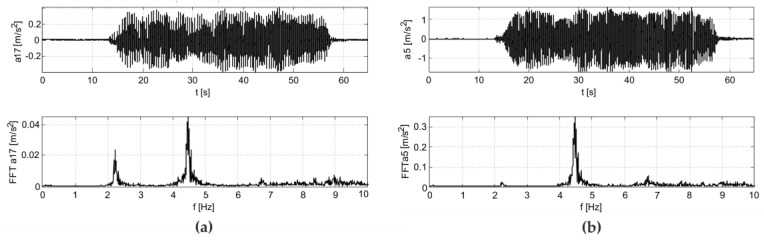
The results of numerical simulations after stand structural tuning from vibrational excitation by the jumping supporters: (**a**) girder, (**b**) roof.

**Figure 26:**
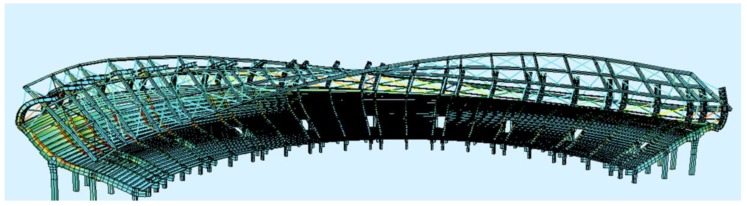
The first numerical form of natural vibrations of the stand after structural tuning.

Figure 25 presents the numerical results obtained during the calculations for the load induced by excitation fans, for the purposes of post-strengthening the stadium structure. The exemplary first form of natural vibration frequencies determined numerically is shown in Figure 26. The dynamic analysis proved that in the range of 2.9 Hz to 5 Hz, there are at least 15 natural vibration frequencies. Such a large number of natural vibration frequencies is ordinary for a spatial structure made of repetitive elements. The strengthening target was to improve the spatial structure performance and reduce natural vibration frequencies within the range of 4.3–4.7 Hz, in order to eliminate the phenomenon of the beat and characteristic of the excitation of two very close natural vibration frequencies.

**Figure 27:**
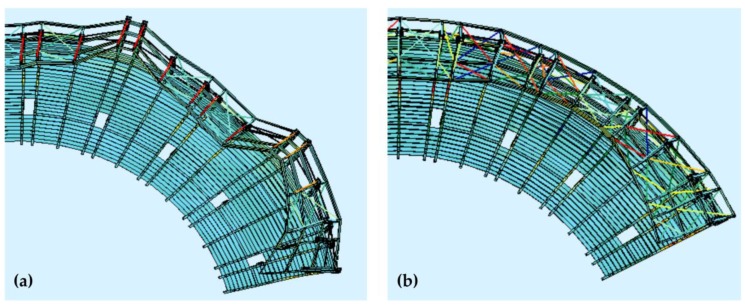
Top view of the stand's roof loaded with synchronous jumps of people showing the vibration amplitudes (**a**) before structural tuning, (**b**) after structural tuning.

Figure 27 shows the visualization of vibrations induced by synchronized dancing of the crowd in the stands, located in rows 22–25 throughout the stadium. The applied dynamic load was a periodic function consisting of a contact phase (sinusoidal impulse) and a flight phase (no load). The load function of a jumping crowd is typically not included in standard regulations. The integration of motion equations was carried out using the Newmark–Wilson method. Figure 27a shows a plane view of the relations of the object degrees of freedom vibration amplitudes in the case when the roof and vertical bracings were fixed in accordance with the design, i.e., every two fields. Figure 27b presents the nature of vibrations for bracings installed in each roofing field. The bracings installation resulted in the roof structure stiffening and in the change of its vibrational spatial character. The calculated amplitude of vibrations from the fans' synchronized dance on the whole structure in rows 22–25 was equal to 3.6 mm at the end of the concrete cantilever, and 5 mm at the free end of the roof. The strengthening method caused a slight reduction in the vibrations of the concrete cantilevers and a significant reduction in the vibration amplitudes of the free end of the roof. A requirement for the correct bracing performance during vibrations was the presence of an initially prestressing force. However, the reduction of concrete cantilevers vibration amplitudes was problematic, because they took over the main part of the dynamic forces generated by fans dancing directly on them.

**Figure 28:**
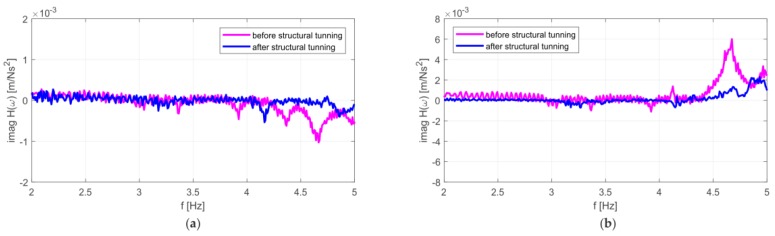
Experimental transition functions for the end of the concrete cantilever (**a**) before and **(b**) after stand structural tuning.

**Figure 29:**
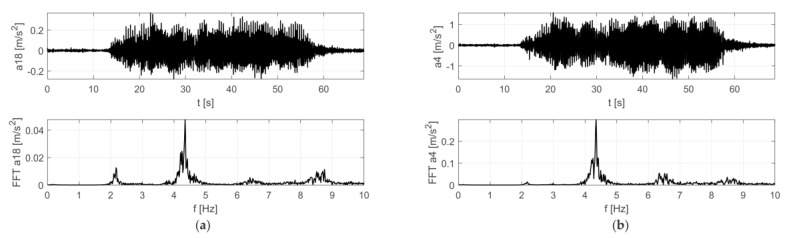
Results of in situ tests after the stand structural tuning from vibration excitation with the jumping supporters: (**a**) girder, (**b**) roof.

Repeated in situ tests were carried out in order to verify the correctness of the original concept of stadium stand tuning. The results of measurements made after structural tuning are shown in Figure 28 and Figure 29. It can be seen that that frequencies identified during crowd-induced vibrations are similar to the measurements before tuning (see Figure 9 and Figure 10), because the same nature of the jumping load. It is important, however, that the natural frequencies of the structure were shifted after structural tuning (see Figure 28), decreasing resonance significantly. The individual resonance zones moved towards the higher frequencies.

## 6. Conclusions

In this study, we adopted the vibration testing method for non-destructive diagnostics of a stadium stand. Experimental and numerical studies were conducted on the real object. The reason for undertaking the investigation was excessive stand vibrations, in particular vertical oscillations of the free end of the roof. On the basis of the conducted vibration tests, the following conclusions of the technical condition of the stadium stand were formulated:

(1) The key natural vibration frequency of 4.68 Hz coincided with the frequency of the higher harmonics of the excitation force (approximately 4.4 Hz).
(2) The reinforced concrete cross-section of the BR2 main girder was scratched. The calculated decrease in the stiffness after scratching was equal to about 50%. The inclusion of scratches reduced the calculated initial natural vibration frequencies of the structure by 5%–10% in comparison with the unscratched cross-section.
(3) The dynamic and synchronic nature of the Labado dance is accidental, but can constitute vandalism. Fans jumping to the rhythm of the animator's regular drumming results in increasing stand vibrations, causing the serviceability limit state to be exceeded. The stand vibrations can lead to material fatigue and, as a result, to its complete destruction.
(4) The proposal to solve the problem of overlapping frequencies causing resonance by changing the animator's drumming rhythm and using it for the needs of dynamic impact elimination in favor of the static impact of people on the stand structure was not accepted by club authorities nor by fans. This objection was explained by the long-term Ladabo dance tradition at the Swiss Krono Arena stadium. Any changes in the drumming rhythm would result in the loss of the cheering spirit among fans used to dancing the Labado.
(5) The proposed original concept of stadium structural tuning significantly reduced the amplitude of the stand structure vibrations. Individual resonance zones were moved towards higher frequencies after tuning, which allowed for safe use of the stadium.
(6) There is a high probability of the occurrence of double natural vibrations frequencies or frequencies with close values in the case of spatial constructions with large dimensions and repeated construction segments. Therein, the phenomenon of the beat will appear, which may cause an increase in tribune elements displacement. The post-tuning FEM model of a structure does not indicate the occurrence of such a case. However, if the actual post-tuning stand structure had double frequencies in a range of up to 5 Hz, additional structure strengthening might be necessary.
(7) The implementation of the architectural structure form resulted in a static scheme in which a roof structure cantilever steel column was mounted at the end of the stand’s reinforced concrete cantilever. The girders working as cantilevers were fixed at the cantilever steel column end. This results in fragility under dynamic load applied to the cantilever part of the concrete beams. Sports facilities must ensure the freedom of supporters gathered in the stands and all possible dances and ways to cheer their favorite teams must be included in the design and implementation process.
(8) The non-destructive tests and numerical simulations conducted in this paper are innovative and pioneering. Currently, no standard has been developed that allows for the determination of performance conditions and methods for testing the resistance of stadium stands to jump loads. The normative documents also lack a ready-to-use jump load function that could be used when designing a structure.

Finally, it can be concluded that the use of the vibration-based method as a non-destructive testing technique for massive structures such as stadiums enabled the creation of a jump function in order to study dance phenomena on the stands. Therefore, it was possible to prevent failures of and damage to the stadium structure, thus ensuring safety for fans supporting their teams. At the design stage, with the use of the created function, the dance influence as a negative factor affecting the stands can be taken into account, allowing the efficient elimination of potential errors and mistakes.
